# Prostate cancer surveillance by occupation and industry: the Canadian Census Health and Environment Cohort (CanCHEC)

**DOI:** 10.1002/cam4.1358

**Published:** 2018-03-01

**Authors:** Jeavana Sritharan, Jill MacLeod, Shelley Harris, Donald C. Cole, Anne Harris, Michael Tjepkema, Paul A. Peters, Paul A. Demers

**Affiliations:** ^1^ Occupational Cancer Research Centre Cancer Care Ontario Toronto Ontario Canada; ^2^ Institute of Medical Science University of Toronto Toronto Ontario Canada; ^3^ Dalla Lana School of Public Health University of Toronto Toronto Ontario Canada; ^4^ School of Occupational and Public Health Ryerson University Toronto Ontario Canada; ^5^ Health Analysis Division Statistics Canada Ottawa Ontario Canada; ^6^ Department of Sociology University of New Brunswick Fredericton New Brunswick Canada

**Keywords:** Cohort, industry, occupation, prostate cancer, surveillance

## Abstract

As there are no well‐established modifiable risk factors for prostate cancer, further evidence is needed on possible factors such as occupation. Our study uses one of the largest Canadian worker cohorts to examine occupation, industry, and prostate cancer and to assess patterns of prostate cancer rates. The Canadian Census Health and Environment Cohort (CanCHEC) was established by linking the 1991 Canadian Census Cohort to the Canadian Cancer Database (1969–2010), Canadian Mortality Database (1991–2011), and Tax Summary Files (1981–2011). A total of 37,695 prostate cancer cases were identified in men aged 25–74 based on age at diagnosis. Cox proportional hazards models were used to estimate hazards ratios and 95% confidence intervals. In men aged 25–74 years, elevated risks were observed in the following occupations: senior management (HR = 1.12, 95% CI: 1.04–1.20); office and administration (HR = 1.19, 95% CI: 1.11–1.27); finance services (HR = 1.09, 95% CI: 1.04–1.14); education (HR = 1.05, 95% CI: 1.00–1.11); agriculture and farm management (HR = 1.12, 95% CI: 1.06–1.17); farm work (HR = 1.11, 95% CI: 1.01–1.21); construction managers (HR = 1.07, 95% CI: 1.01–1.14); firefighting (HR = 1.17, 95% CI: 1.01–1.36); and police work (HR = 1.22, 95% CI: 1.09–1.36). Decreased risks were observed across other construction and transportation occupations. Results by industry were consistent with occupation results. Associations were identified for white‐collar, agriculture, protective services, construction, and transportation occupations. These findings emphasize the need for further study of job‐related exposures and the potential influence of nonoccupational factors such as screening practices.

## Introduction

Prostate cancer is one of the most commonly diagnosed cancers worldwide and accounts for 15% of all cancers diagnosed in men 1, 2. It is more common in men over the age of 50, but in recent years, it has been diagnosed with increased frequency in younger men 2. Through efforts to understand the etiology of prostate cancer, the most well‐established risk factors are age, family history of prostate cancer, and ethnicity 3. Other factors of diet, obesity, smoking, sexual behavior, sexually transmitted diseases, genetic mutations, hormone levels, and occupation have shown mixed evidence 3, 4, 5. There are currently no established occupational risk factors for prostate cancer; however, the International Agency for Research on Cancer (IARC) has concluded there is limited evidence for arsenic and cadmium compounds, the insecticide malathion, radiation, and the rubber production industry 6. Other associations have also been observed for agriculture occupations, firefighting occupations, shift work, and whole‐body vibrations 7, 8, 9, 10, 11, 12, 13, 14, 15.

Examining disease risks across occupational and industry groups can lead to better understanding of associated occupational exposures 16. Occupation and industry groups can act as surrogates for exposure and indicative of where prevention research should focus. In recent years, large international cohort studies examined prostate cancer risk across a range of occupation and industry groups and observed inconsistent associations 17, 18, 19. These cohort studies observed associations with white‐collar occupations suggesting these workers may have better access to screening 17, 18, 19. Further understanding of occupation and screening behaviors is needed. It is well established that prostate cancer incidence has increased over time and that this increase can be accounted for primarily because of prostate‐specific antigen (PSA) screening 20. However, it is still unclear how screening patterns affect changes in incidence within an occupational group. Given the current uncertainty regarding the benefits of early diagnosis of prostate cancer using PSA screening 21, it is important to strengthen the evidence on preventative factors, such as occupation. This will not only confirm existing associations and generate new hypotheses, but will also provide better understanding of how associations between occupation and prostate cancer are influenced by screening‐related factors.

The 1991 Canadian Census Health and Environment Cohort (CanCHEC) is one of the largest population‐based cohort studies in Canada spanning across all provinces and territories 22. This cohort provides unique linked data that contain valuable information on occupation and prostate cancer. This study provides national‐level data with a large sample size with detailed information on occupational and nonoccupational factors measured at baseline 23. The purpose of this study was to evaluate prostate cancer by occupation and industry employment in the 1991 CanCHEC.

## Materials and Methods

### Study population and linkage

**Figure 1:**
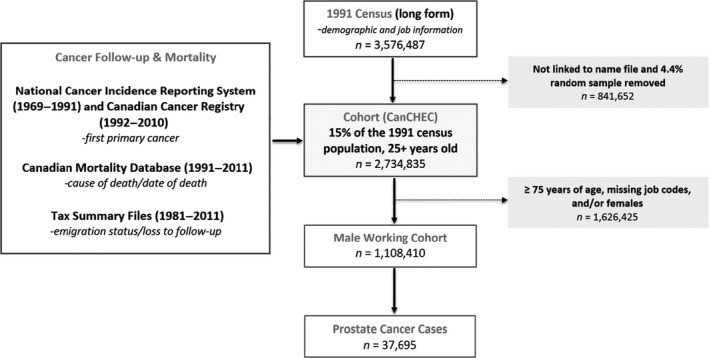
Flowchart illustrating the Canadian Census Health and Environment Cohort (CanCHEC) linkage and the number of prostate cancer cases derived from the working cohort.

The CanCHEC (*n* = 2,743,835) was established by Statistics Canada linking data from the long‐form 1991 Canadian Census to the Canadian Cancer Database (1969–2010), Canadian Mortality Database (1991–2011), and annual Tax Summary Files (1981–2011) (Fig. 1). A detailed description of the linkage methodology is published elsewhere 22, 23, and a brief flowchart of the linkage is shown in Figure 1. The mandatory 1991 Canadian long‐form census questionnaire was administered to 20% of Canadian households on 4 June 1991. Individuals included in the cohort were 25 years or older on census day, noninstitutional residents, and had filed taxes in 1991 or 1992 22, 23. The Canadian Cancer Database provided cancer morbidity information and was prepared from data received by the Canadian Cancer Registry (1992–2010) and the National Cancer Incidence Reporting System (1969–1991). The Canadian Mortality database provided information on cause of death and date of death. Residential, marital status, and loss to follow‐up information were identified using the Tax Summary Files. Loss to follow‐up was determined if individuals emigrated out of Canada or if they did not file income taxes for over four consecutive years. Follow‐up of all individuals began on census day, 4 June 1991, and continued until the end of follow‐up on 31 December 2010 or until date of prostate cancer diagnosis, date of death, or loss to follow‐up 22, 23.

### Work history

In the 1991 long‐form census, respondents were asked to report on the occupation they held in the week prior to the census. If no job was held in the week prior, respondents were asked for the job of longest duration since 1 January 1990. If more than one job was held in the week prior to the census, respondents answered based on the job in which the most hours were worked 22, 23. Job information from each individual was then coded to occupation and industry classification codes using the 1991 Standard Occupation Classification (SOC91) and 1980 Standard Industrial Classification (SIC80). We then used the four‐digit codes from SOC91 and SIC80 to obtain the most descriptive job titles, and we categorized workers based on similar job titles and tasks (related to potential exposures) to ensure they were in the appropriate occupation and industry groups for analysis. The working cohort included 2,051,315 individuals 22, 23. For this study, the cohort was restricted to only men (*n* = 1,108,410), aged 25–74 years (at baseline) who had a valid entry for occupation in the 1991 long‐form census 22, 23. The valid entry for occupation was based on if they reported an occupation in the census questionnaire—the job they held in the week prior to the census, longest held since the year prior, or the job they worked the most hours in. Individuals who did not list an occupation were excluded from the working cohort.

### Prostate cancer diagnosis

Prostate cancer was the primary interest of this study, and each prostate cancer case was defined as an incident diagnosis between 1992 and 2010 based on information from the Canadian Cancer Database. This database provided information on cancer diagnoses going back to 1969 to capture cases prior to 1992 and to confirm that each case included in this study was a primary prostate cancer diagnosis. Year of death was obtained from the Canadian Mortality Database to remove any deceased individuals from the cohort and to make sure that the prostate cancer diagnosis preceded the death date. The mortality database provided cause of death information to identify prostate cancer‐related deaths. Cancer cases were classified according to the 9th revision of the International Classification of Diseases (ICD‐9) and 3rd revision of the International Classification of Diseases for Oncology (ICD‐O‐3) 22, 23.

### Statistical analysis

Hazard rate ratios (HRs) and corresponding 95% confidence intervals (CI) were calculated using Cox proportional hazard regression models to estimate prostate cancer risks associated with employment by occupation and industry. Men not employed in the specific occupation or industry being evaluated served as the nonexposed reference group. Prostate cancer is more common in men over the age of 50 years as risk of prostate cancer significantly increases with older age and these men are more likely to get screened for prostate cancer. However, younger diagnoses for prostate cancer (>50 years) are rare, likely to be aggressive forms, or from genetic susceptibility, and younger men are less likely to get screened 2. These factors make it difficult to capture a large number of prostate cancer cases in younger men (<50 years) in population studies, and our study is unique in that it had the ability to capture both younger and older prostate cancer cases. We originally analyzed the data with two age‐groups (25–49 years and 50–74 years, Tables S2 and S3) but given that there were smaller case counts in the younger men and there were no meaningful differences in findings between the two age‐groups, we decided to use the combined age‐group of men 25–74 years for the final analysis. The primary focus of this analysis was prostate cancer incidence, with an additional analysis on mortality from prostate cancer (data not shown). All HR estimates were adjusted for baseline covariates of age, province of residence, ethnicity, education, and marital status—all of which were obtained from the 1991 Census. Income adjustment showed less than a 10% change in HR estimates and therefore was removed from the model. Age‐standardized prostate cancer rates were also examined for the working overall cohort and specific occupation groups, standardized to the 2001 CanCHEC population. In accordance with Statistics Canada disclosure rules, case counts of less than five were not included in reported tables and all frequencies were rounded to the nearest 100. All statistical analyses were performed using SAS 9.4 (SAS Institute, Cary, NC) and took place in the secure facility of the Statistics Canada Research Data Centre in Toronto.

### Study approval

The linkage was approved by the Statistics Canada Executive Management Board, and this study was approved by the Statistics Canada Research Data Centre and the University of Toronto Health Sciences Research Ethics Board.

## Results

A total of 37,695 incident prostate cancer cases and 1700 deaths from prostate cancer were identified between 1992 and 2010 in the overall working cohort of men aged 25–74 years (*n* = 1,108,410) (Fig. 1). Table 1 presents the baseline characteristics of prostate cancer cases. An increased risk of prostate cancer was observed among black men when compared to Caucasian men (HR = 1.77, 95% CI: 1.66–1.89; fully adjusted). Men in other ethnic groups had reduced risks when compared to Caucasian men. Prostate cancer risks increased with increasing level of education. Decreased risks were observed among men who were never married or separated/divorced/widowed when compared to men who were legally married/common law.

**Table 1:** Baseline characteristics of the working cohort and of men with prostate cancer in the CanCHEC (Ages 25–74 years)

	All workers (%) (*n* = 1,108,410)	Workers with PC (%) (*n* = 37, 695)	Workers with PC HR^a^ (95% CI)
Age‐Group			
25–34	359, 075 (32.4)	765 (2.0)	
35–44	341, 515 (30.8)	5, 885 (15.6)	
45–54	229, 460 (20.7)	13, 285 (35.2)	
55–64	143, 895 (13.0)	14, 045 (37.3)	
65–74	34, 465 (3.1)	3, 720 (9.9)	
Province of residence			
Ontario	404, 130 (36.5)	15, 605 (41.4)	
Quebec	276, 120 (24.9)	6, 195 (16.4)	
Manitoba	47, 375 (4.3)	1, 585 (4.2)	
Saskatchewan	42, 050 (3.8)	1, 825 (4.8)	
Alberta	107, 405 (9.7)	4, 035 (10.7)	
British Columbia	130, 815 (11.8)	4, 965 (13.2)	
Yukon, NWT, Nunavut	11, 395 (1.0)	225 (0.6)	
Newfoundland	21, 815 (2.0)	680 (1.8)	
Prince Edward Island	4, 945 (0.4)	210 (0.6)	
Nova Scotia	34, 750 (3.1)	1, 245 (3.3)	
New Brunswick	27, 600 (2.5)	1, 130 (3.0)	
Ethnicity			
Caucasian	1, 018, 990	35, 345 (93.8)	Ref
Black	15, 120	910 (2.4)	**1.77 (1.66–1.89)**
South/East/South‐East Asian/Pacific Islander	58, 100	1, 120 (3.0)	**0.54 (0.51–0.57)**
Southwest Asian/Arabic	10, 850	235 (0.6)	**0.72 (0.63–0.82)**
Latin American	3, 925	55 (0.2)	**0.67 (0.52–0.88)**
Other, Multiple	1, 425	35 (0.1)	0.93 (0.68–1.28)
Highest level of education completed			
No High School	Ref	13, 090 (34.7)	Ref
High School	444, 560 (40.1)	13, 430 (35.6)	**1.06 (1.03–1.08)**
Postsecondary Nonuniversity/Trade School	154, 165 (13.9)	4, 375 (11.6)	**1.10 (1.06–1.14)**
University Degree	187, 495 (16.9)	6, 805 (18.1)	**1.22 (1.19–1.26)**
Marital status			
Legally Married/Common Law	152, 205	33, 770 (89.6)	Ref
Never Married	92, 435	1, 550 (4.1)	**0.75 (0.71–0.79)**
Separated/Divorced/Widowed	63, 770	2, 380 (6.3)	**0.92 (0.88–0.96)**
Broad occupational groups			
(A) Management	158, 105	6, 620	**1.07 (1.04–1.10)**
(B) Business, Finance, and Administrative	98, 265	3, 340	**1.04 (1.00–1.08)**
(C) Natural, Applied Sciences, and Related	85, 390	2, 470	0.99 (0.95–1.03)
(D) Health	22, 575	805	0.99 (0.92–1.07)
(E) Social Science, Education, Government Service, and Religion	64, 525	2, 715	1.00 (0.96–1.05)
(F) Art, Culture, Recreation, and Sport	19, 560	555	0.98 (0.90–1.07)
(G) Sales and Services	174, 795	5, 835	1.01 (0.98–1.04)
(H) Trades, Transport, Equipment Operators, and Related	300, 690	9, 020	0.92 (0.90–0.95)
(I) Occupations Unique to Primary Industry	78, 010	3, 445	**1.08 (1.04–1.12)**
(J) Occupations Unique to Processing, Manufacturing, and Utilities	106, 495	2, 895	0.95 (0.91–0.98)
Person‐years of follow‐up	19, 635, 045	463, 760	
Mean person‐years of follow‐up	17.7	12.0	

PC, prostate cancer.

a adjusted for age, province, ethnicity, education, and marital status.

Case counts are rounded to base 5 using random rounding.

Bold values represent statistically significant hazard ratios (*p*<0.05)

**Figure 2:**
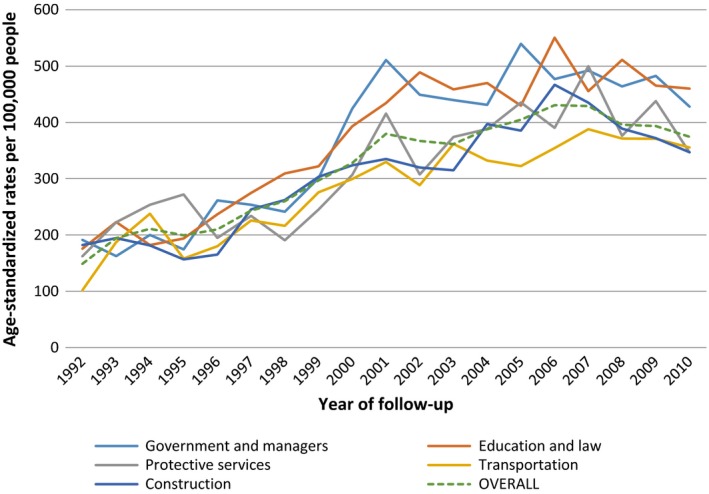
Age‐standardized prostate cancer rates by year for specific occupation groups and for the overall working cohort. The solid lines represent major occupation groups of government and managers, education and law, protective services, transportation, and construction. The dashed line represents the overall working cohort. All prostate cancer rates were standardized to the 2001 CanCHEC population to account for differences in age structure each year and to allow for comparability of rates each year.

Prostate cancer by selected occupations is shown in Table 2 and by selected industries in Table S1. Figure 2 shows prostate cancer rates for specific occupation groups and for the overall working cohort.

**Table 2:** Hazard Ratios (HR) and Confidence Intervals (CI) by Occupation Group in the CanCHEC (Ages 25–74 years)

Occupation Groups	Number of PC cases (*n* = 37, 695)	Number of noncases (*n* = 1,070,715)	HR^a^ (95% CI)
Administrative and related			
Senior and Government Managers	755	13,505	**1.12 (1.04–1.20)**
Office Managers	820	17,285	1.00 (0.96–1.05)
Other Office and Administration	2120	63,705	**1.19 (1.11–1.27)**
Finance Managers and Financial Services	1860	45,935	**1.09 (1.04–1.14)**
Legal Services and Related	300	6995	1.00 (0.89–1.12)
Education Instructors and Related	2030	43,795	**1.05 (1.00–1.11)**
Natural Resources			
Agriculture/Farm Managers and Supervisors	1945	29,445	**1.12 (1.06–1.17)**
Agricultural Specialists and Technicians	120^b^	3435	1.04 (0.87–1.24)
General Farm Workers and Laborers	475	11,120	**1.11 (1.01–1.21)**
Logging Operators and Laborers	50^b^	2100	0.92 (0.70–1.20)
Forestry Technicians and Professionals	320^b^	11,000	1.04 (0.93–1.16)
Fishing Laborers, Trapping, and Hunting	260	6995	1.00 (0.88–1.13)
Mining Production and Laborers	195^b^	6975	0.89 (0.77–1.02)
Primary Production, Transportation, and Manufacturing Managers	645	13,955	**1.11 (1.03–1.20)**
Woodworking, Carpentry, and Processing; Sawmill	995	32,480	0.95 (0.89–1.01)
Pulp and Paper Mill Machine Operators	190^b^	8215	0.90 (0.78–1.04)
Metal Processing, Machinery, and Construction			
Metal and Mineral Processing	750	23,515	0.96 (0.89–1.03)
Machinists and Tool Operators	405	12,065	1.01 (0.92–1.11)
Machine Assemblers and Manufacturers	340	10,410	1.05 (0.94–1.17)
Rubber and Plastic Products	100^b^	4750	0.88 (0.72–1.06)
Plumbers, Pipefitters, and Gas fitters	340^b^	10,095	0.98 (0.88–1.09)
Painters	185	6060	0.95 (0.82–1.10)
Construction Managers and Supervisors	1125	24,015	**1.07 (1.01–1.14)**
Construction Trades	720	26,830	0.89 (0.83–0.96)
Transportation and Related			
Transportation Equipment Operators	925	27,180	0.91 (0.85–0.97)
Transportation Technicians and Maintenance Workers	285	8900	1.01 (0.89–1.13)
Motor Vehicle Repairers	545	23,135	0.87 (0.80–0.95)
Vehicle Drivers	1605	51,270	0.92 (0.87–0.97)
Protective Services			
Firefighters	165^b^	4365	**1.17 (1.01–1.36)**
Armed Forces	200	8965	1.10 (0.95–1.26)
Police Officers	325	9730	**1.22 (1.09–1.36)**
Other Protection Services	565^b^	12,435	0.97 (0.90–1.04)
Health and Personal Care			
Dentists and Related	120	2585	1.08 (0.91–1.30)
General and Specialist Physicians	305^b^	6295	0.92 (0.82–1.03)
Registered Nurses, Supervisors, and Aides	120	5130	0.98 (0.82–1.17)
Other Health Professionals and Related	595	16,275	1.02 (0.94–1.11)

PC, prostate cancer.

a Hazard ratios (HR) adjusted for age, province, ethnicity, education, and marital status; Reference group: men employed in all other occupations except the occupation of interest.

b Missing ±5 to 10 cases because of low case counts in younger age categories.

All case counts are rounded to base 5 using random rounding and counts <5 are not shown as per Statistics Canada reporting guidelines.

Bold values represent statistically significant hazard ratios (*p*<0.05)

### Administrative and management

Significant elevated risks were observed across most administrative and management occupations for both men aged 25–74 years. Significant elevated risks were observed for senior managers (HR = 1.12, 95% CI: 1.04–1.20), office managers (1.19, 95% CI: 1.11–1.27), finance service occupations (1.09, 95% CI: 1.04–1.14), and education service occupations (1.05, 95% CI: 1.00–1.11). Elevated risks were also observed for office (nonmanagerial) and legal service occupations, although these results were not significant. Industry findings were similar to occupation findings, with elevated risks across administrative jobs. No statistically significant associations were observed for prostate cancer mortality.

### Natural resources

Significant elevated risks were observed in occupations of agriculture/farm managers and supervisors (HR = 1.12, 95% CI: 1.06–1.17), farm workers and laborers (HR = 1.11, 95% CI: 1.01–1.21), and primary production/transportation/manufacturing managers (HR = 1.11, 95% CI: 1.03–1.20). Nonsignificant elevated risks were observed for forestry, fishing and trapping, and woodworking occupations. Industry findings also showed a significant elevated risk for agriculture industries (1.11, 95% CI: 1.06–1.16), with nonsignificantly elevated risks across other natural resource‐based work. With mortality, a statistically significant association was observed for agricultural managers (HR = 1.42, 95% CI: 1.21–1.68).

### Protective services

Significant elevated risks were observed across protective services occupations for firefighters (HR = 1.17, 95% CI: 1.01–1.36) and police officers (HR = 1.22, 95% CI: 1.09–1.36), and a nonsignificant elevated risk was observed for armed forces (HR = 1.10, 95% CI: 0.95–1.26) and other protective services (0.97, 95% CI: 0.90–1.04). Protective services are categorized under government services at an industry level and cannot be grouped separately; however, the government–industry groups were observed to be elevated. No statistically significant associations were observed for prostate cancer mortality.

### Construction, transportation, and other

A significant elevated risk was observed for construction managers (HR = 1.07, 95% CI: 1.01–1.14), with significant decreased risks for construction trades (HR = 0.89, 95% CI: 0.83–0.96), transportation equipment operators (HR = 0.91, 95% 0.85–0.97), motor vehicle repairers (HR = 0.87, 95% CI: 0.80–0.95), and vehicle drivers (HR = 0.92, 95% CI: 0.87–0.97). Nonsignificantly elevated risks were observed for electrical assemblers, electricians and electrical trade, and transportation technologists and technicians. Industry results were similar to occupation results for construction and transportation workers, showing mixed findings overall. No statistically significant associations were observed for prostate cancer mortality. By industry, there were additional statistically significant findings for utility services and telecommunications industries (Table S1).

### Prostate cancer rates by occupation

Figure 2 presents the prostate cancer rates for specific occupation groups and for the overall CanCHEC working cohort. Government, management, education, and law occupations had higher prostate cancer rates than the overall working cohort, whereas construction and transportation occupations had lower prostate cancer rates than the overall working cohort. The rates for protective services occupations were similar to the overall working cohort rate.

## Discussion

In this large cohort study, significant associations with prostate cancer risk were observed for ethnicity, education, and marital status. Based on primarily the United States studies, risk of prostate cancer is known to be highest among Black/African American men than any other race, followed by Caucasian men 24, 25. Men of other ethnic groups are recognized as having reduced risks of prostate cancer 24, 25. Differences by ethnicity may be influenced by dietary differences, genetic predisposition, socioeconomic factors, access to quality care, and disparities in screening and diagnosis 26, 27. The elevated risks observed in men with higher education can be interpreted with socioeconomic status. Men with higher socioeconomic status (SES) may have better access to health care and screening resources leading to early diagnosis, whereas men with lower SES may face more barriers to accessing screening and medical facilities 19, 28. In Canada, it is unclear to what extent healthcare accessibility is affected by differences in SES, as universal healthcare coverage has been shown to reduce inequalities in different SES groups 29. Also, married men are more likely to utilize shared decision‐making with their partner and may be influenced by their partner to seek better health behaviors than men who are not married 30, 31. It has also been shown that if married men have a family history of prostate cancer, they are more likely to get screened 32. We were unable to assess family history of prostate cancer in our study. Factors related to family physician visits and medical history can also affect frequency of screening; however, we did not have this information available. All of these factors are related to screening behaviors, and to account for potential screening bias, we adjusted hazard ratios for these factors. Age was also included in the adjustments as it is well established that the risk of prostate cancer increases with increased age, and screening is more likely in older men 3, 33. Some of the results attenuated, but overall, there was a less than 10% change in hazard ratios. With screening bias, it is asserted that increased screening leads to increased incidence and reduced mortality over time. In this study, we observed no associations between employment in white‐collar work and prostate cancer mortality, which supports screening bias.

Our study found consistent elevated risks for jobs in administration and related occupations and industries. These types of jobs included workers in government, senior management, office, business, finance, law, and education. Some studies have previously shown similar elevated risks in men employed in white‐collar jobs 19, 34, 35, 36, 37. As white‐collar jobs are recognized as having few chemical exposures, findings may reflect other factors of physical activity, socioeconomic factors, and screening. Men employed in these occupations are likely to have sedentary work environments with lower levels of occupational physical activity 35. Although physical activity could not be assessed in our study, lower levels of physical activity may be linked to androgen metabolism and reduced immune responses failing to prevent tumor formation 35. Many of these white‐collar jobs are typically associated with higher education and income which is often associated with informed decision‐making and better accessibility to health resources, including prostate cancer screening 19. When looking at education and income levels and men with prostate cancer in white‐collar jobs, we observed a nonsignificantly elevated risk for senior management workers who had a university degree (highest education category). However, this was not statistically significant and we did not see any other associations related to education or income across white‐collar or administrative jobs. Based on our findings, further evidence is needed on physical activity and screening‐related factors and patterns in white‐collar and administrative workers to better understand how these factors are involved in prostate cancer risk.

Men employed in agriculture were at increased risk of prostate cancer diagnosis and mortality, which is consistent with previous literature 8, 9, 10. Exposure to pesticides and diesel engine exhaust from farm equipment is suspected contributing risk factors. Exposure to pesticides may affect hormone levels and function by disrupting endocrine activity and increasing estrogen levels leading to tumor promotion 38, 39, 40. The Agriculture Health Study (AHS) has consistently reported elevated risks for prostate cancer among pesticide applicator occupations with mixed findings for specific pesticides linked to prostate cancer 38, 39, 40, 41, 42, 43. Family history of prostate cancer is also recognized as a potential modifier for specific pesticide exposures such as fonofos and aldrin and prostate cancer risk, but not for other pesticide exposures 38, 39, 40, 41, 42, 43. We observed a reduced risk in the mining industry across both age‐groups, which is consistent with previous findings 44. Few studies in the past have examined prostate cancer risk in natural resource‐based jobs, aside from agriculture, and these studies observed mixed findings 14, 34, 45. Given the limited evidence, further investigation into these natural resource‐based occupations is needed to understand what chemical exposures or lifestyle factors are involved.

Significantly elevated risks were observed for firefighters and police officers, with nonsignificantly elevated risk in armed forces and other protective services. Protective services occupations involve exposure to diesel exhaust, dust and particulate matter, chemical agents, radiation, and other mixed agents. They may also experience disruption of the circadian rhythm from shift work, and they can be under constant psychological stress which may impact biological processes leading to the development of cancer 7, 46, 47. Specifically, police officers may spend extensive periods driving or near vehicles which can lead to increased exposure to vehicle exhaust 46. A recent Montreal case–control study reported a similar elevated risk in police officers 34. Firefighters, in a highly hazardous job, are exposed to fires that release carcinogenic substances and toxins 47. Potential exposures in firefighting include mixtures of particulate, gases and fumes, diesel exhaust, and polychlorinated biphenyls 10, 48, 49. Based on existing evidence, IARC classified firefighting as possibly carcinogenic to humans (Group 2B) 50. Screening may also be an important factor in firefighting occupations. It is speculated that there may have been targeted screening in firefighters in the 1990s; however, there is no documentation available on screening by occupation for this time period 48. For men in the armed forces, we observed a nonsignificantly elevated risk, although other international studies have found significant elevated risks 51, 52. Men in the armed forces are involved in high‐risk environments and are exposed to many different types of agents. They are also more likely to get screened compared to men in other jobs. Specifically, the Canadian national defense and armed forces require frequent health examinations which may lead to better access to health resources and screening 53. Given our findings and other recent evidence on prostate cancer risk in protective services, it is necessary to understand and compare job‐specific exposures in each individual job (firefighting, police, armed forces) while also determining the impact of screening or availability of health resources in these jobs.

Few elevated risks were observed in occupations of construction and transportation. Previous studies have reported elevated risks associated with employment in construction and transportation, with some evidence linked to whole‐body vibrations, diesel exhaust, and polycyclic aromatic hydrocarbons (PAH) 12, 13, 14, 15. However, some studies have also reported mixed results across construction and transportation workers similar to our findings 18, 54, 55. Our findings could be related to differences in prostate cancer screening in these jobs. A recent presentation identified that men in construction and transportation jobs were less likely to get screened than men in management jobs 56.

Based on our findings in occupation groups, we were interested in understanding if prostate cancer rates in the discussed occupations groups were similar to that of prostate cancer rates in Canadian men over the time period of the cohort. Across most provinces in Canada, prostate cancer incidence began to accelerate in 1990 and marked peaks of incidence were observed in both 1993 and 2001, followed by a steady decline 20. This pattern is aligned with the introduction of PSA testing in the 1990s and a surge of PSA testing and overdiagnosis in 2001 in Canada 20. We investigated if observed prostate cancer rates across specific occupations in our study showed similar patterns to the trend recognized across Canada. Occupations of government/management and education/law showed peaks that were during similar time periods as recognized across Canada (Fig. 2). Observed prostate cancer rates in these occupations were also higher than the overall 1991 working cohort. These findings may be attributed to increased screening behaviors leading to increased diagnosis of prostate cancer among these workers. Protective services occupations show few peaks during the time of increased PSA testing and during other time periods (Fig. 2). It is difficult to determine whether the rates observed in protective services are due to screening or other factors. Observed prostate cancer rates in construction and transportation occupations were lower than the overall working cohort, and there were no identified prominent peaks during the time of increased screening (Fig. 2). Construction and transportation workers may have decreased screening activity leading to fewer prostate cancer cases identified; however, further evidence is needed. Future studies should compare risk of prostate cancer in blue‐collar work and white‐collar work, while evaluating actual screening rates in these jobs.

Increased prostate cancer screening has also shown to reduce mortality from prostate cancer 20. Occupations related to white‐collar work, protective services, and construction and transportation showed no association with prostate cancer mortality in this study, whereas agriculture work was found to have an elevated risk of mortality. This may be indicative of screening differences by type of occupation, specifically showing that there may be increased prostate cancer screening in white‐collar and protective services jobs, and a lack of screening in agricultural workers. However, interpretation of findings is limited and further investigation into mortality from prostate cancer and occupation is needed.

Our study has some limitations and strengths. It only contains employment information at one point in time in 1991 and does not have data on employment duration. It also lacks information on family history of prostate cancer and physical activity, which may act as confounders or effect modifiers. There was no information on screening behaviors, although we accounted for factors related to screening. This study also had no information on aggressiveness of prostate cancer cases, but our analysis was able to capture cases in younger men which are usually rare and likely to be aggressive forms. Also, this study makes multiple comparisons which can lead to some chance findings. There are also distinct strengths in this study. It is one of the largest Canadian cohorts with information on occupation and prostate cancer, and other nonoccupational factors. The large sample size provided the ability to detect a large number of prostate cancer cases with increased power and less likelihood of type I error. The large sample also captured cases under the age of 50 years which is generally difficult to obtain.

In this study, we observed elevated risks of incidence in jobs related to administration and management, agriculture, and protective services and decreased risks in construction and transportation in men aged 25–74 years. Also, an elevated risk of mortality was observed in agriculture management workers. Findings show that there may be different factors involved such as job‐specific exposures, lifestyle factors, and screening behaviors. Future studies should focus on the identified occupation groups in this study to pinpoint job‐specific exposures while reporting on the screening behaviors of these workers. Further investigation is also needed on occupation and aggressive forms of prostate cancer, especially in younger age‐groups. This will provide better direction on the relationship between occupation, related exposures, and screening patterns.

## Study Approval

The linkage was approved by the Statistics Canada Executive Management Board, and this study was approved by the Statistics Canada Research Data Centre and the University of Toronto Health Sciences Research Ethics Board.

## Ethical Approval

Ethics approval was granted by the University of Toronto (#26517).

## Conflict of Interest

The authors declare that they have no conflict of interest.

## Supporting information


**Table S1**. Hazard Ratios (HR) and Confidence Intervals (CI) by Industry Group in the CanCHEC (Ages 25–74 years).
**Table S2**. Hazard Ratios (HR) and Confidence Intervals (CI) for Prostate Cancer by Occupation Group in the CanCHEC, stratified by age.
**Table S3**. Hazard Ratios (HR) and Confidence Intervals (CI) for Prostate Cancer by Industry Group in the CanCHEC, stratified by age.
