# Supplementary material for: Prostate cancer surveillance by occupation and industry: the Canadian Census Health and Environment Cohort (CanCHEC)
Source: Cancer Med. 2018 Mar 1;7(4):1468–78. doi: 10.1002/cam4.1358 (PMC5911573; doi:10.1002/cam4.1358)
Supplement: Supplementary file 1 — Table S1. Hazard Ratios (HR) and Confidence Intervals (CI) by Industry Group in the CanCHEC (Ages 25–74 years). Table S2. Hazard Ratios (HR) and Confidence Intervals (CI) for Prostate Cancer by Occupation Group in the CanCHEC, stratified by age. Table S3. Hazard Ratios (HR) and Confidence Intervals (CI) for Prostate Cancer by Industry Group in the CanCHEC, stratified by age. [file CAM4-7-1468-s001.docx]

| **Supplementary Table 1. Hazard Ratios (HR) and Confidence Intervals (CI) by Industry Group in the CanCHEC (Ages 25-74 years)** | | | |
| --- | --- | --- | --- |
| **Industry Groups** | **Number of PC cases  (n=37, 695)** | **Number of non-cases (n=1,070,715)** | **HR^a^ (95% CI)** |
| **Government and Administrative** |  |  |  |
| Federal Government Services | 1435 | 37870 | 1.11(1.05-1.17) |
| Provincial and Territorial Government Services | 1020 | 26005 | 1.04(0.97-1.10) |
| Local and Other Government Services | 1250 | 37520 | 0.96(0.91-1.02) |
| Business Services | 2300 | 60370 | 1.02(0.98-1.06) |
| Finance Services | 1055 | 27110 | 1.13(1.06-1.20) |
| Educational Services | 2645 | 58590 | 1.02(0.98-1.06) |
| **Natural Resources** |  |  |  |
| Agriculture Industries | 2545 | 44430 | 1.11(1.06-1.16) |
| Services Incidental to Agriculture | 70* | 1890 | 1.05(0.83-1.32) |
| Forestry Services | 95* | 3640 | 0.99(0.82-1.21) |
| Logging Services | 310* | 10285 | 0.94(0.84-1.05) |
| Fishing and Trapping | 250 | 6730 | 1.02(0.90-1.15) |
| Wood Products | 465 | 16315 | 0.96(0.87-1.05) |
| Paper and Allied Products | 580 | 16625 | 1.04(0.96-1.13) |
| Mining (Metal, Non-Metal, Coal Mines) | 465 | 15945 | 0.88(0.80-0.96) |
| **Metal and Non-Metal Products** |  |  |  |
| Primary Metal Products | 590 | 16430 | 0.98(0.90-1.06) |
| Fabricated Metal Products | 625 | 18515 | 1.03(0.95-1.11) |
| Non-Metal Mineral Products | 275* | 7360 | 1.04(0.93-1.18) |
| Plastic Products | 200* | 6285 | 1.12(0.98-1.29) |
| Rubber Products | 75* | 2870 | 0.88(0.70-1.10) |
| Chemical Products | 285 | 8885 | 0.98(0.88-1.11) |
| **Transportation and Trades** |  |  |  |
| Transportation Equipment and Repair | 1130 | 33845 | 0.98(0.93-1.04) |
| Service Industries Incidental to Transportation | 325 | 58315 | 1.01(0.97-1.06) |
| Other Transportation | 2070 | 10080 | 0.86(0.77-0.96) |
| Automotive Vehicles, Parts and Accessories | 1080 | 38305 | 0.96(0.90-1.02) |
| **Construction** |  |  |  |
| General Contracting and Development | 1000 | 31690 | 0.96(0.91-1.03) |
| Trade Contracting and Site Work | 1645 | 55315 | 0.93(0.88-0.98) |
| Heavy Industrial Construction | 420 | 12655 | 0.93(0.85-1.02) |
| Service Industries Incidental to Construction | 340 | 9320 | 1.03(0.93-1.15) |
| **Technical and Informational Industries** |  |  |  |
| Telecommunications | 930 | 27770 | 1.11(1.04-1.18) |
| Other Utility Industries (Electric, Gas, Water) | 695 | 18955 | 1.10(1.02-1.19) |
| Printing, Publishing, and Allied Industries | 430 | 13775 | 0.96(0.87-1.05) |
| **Health Service Industries** |  |  |  |
| Health and Social Services | 1260 | 36690 | 0.96(0.91-1.01) |
| *PC – prostate cancer ^a^Hazard ratios (HR) adjusted for age, province, ethnicity, education, and marital status; Reference group: men employed in all other industries except the industry of interest *Missing +/- 5 to 10 cases because of low case counts in younger age categories*  *All case counts are rounded to base 5 using random rounding and counts <5 are not shown as per Statistics Canada reporting guidelines* | | | |

| **Supplementary Table 2. Hazard Ratios (HR) and Confidence Intervals (CI) for Prostate Cancer by Occupation Group in the CanCHEC, stratified by age** | | | | |  |
| --- | --- | --- | --- | --- | --- |
|  | **Age 25-49 years** | | **Age 50-74 years** | | |
| **Occupation Groups** | **Cases** | **HR^a^ (95% CI)** | **Cases** | **HR^a^ (95% CI)** | |
| **Administrative and Related** |  |  |  |  | |
| Senior and Government Managers | 10 | **1.12 (1.04-1.20)** | 745 | **1.09 (1.02-1.17)** | |
| Office Managers | 10 | **1.19 (1.11-1.27)** | 810 | 1.00 (0.96-1.04) | |
| Other Office and Administration | 30 | 1.00 (0.96-1.05) | 2090 | **1.16 (1.08-1.24)** | |
| Finance Managers and Financial Services | 35 | **1.09 (1.04-1.14)** | 1825 | **1.08 (1.03-1.13)** | |
| Legal Services and Related | 5 | 1.00 (0.89-1.12) | 295 | 1.01 (0.90-1.13) | |
| Education Instructors and Related | 25 | **1.05 (1.00-1.11)** | 2005 | 0.99 (0.94-1.04) | |
| **Natural Resources** |  |  |  |  | |
| Agriculture/Farm Managers and Supervisors | 15 | **1.12 (1.06-1.17)** | 1930 | **1.11 (1.06-1.17)** | |
| Agricultural Specialists and Technicians | - | - | 120 | 1.04 (0.87-1.24) | |
| General Farm Workers and Labourers | 5 | **1.11 (1.01-1.21)** | 470 | **1.12 (1.02-1.23)** | |
| Logging Operators and Labourers | - | - | 50 | 0.94 (0.72-1.24) | |
| Forestry Technicians and Professionals | - | - | 320 | 1.09 (0.97-1.21) | |
| Fishing Labourers, Trapping and Hunting | 5 | 1.00 (0.88-1.13) | 255 | 1.02 (0.90-1.15) | |
| Mining Production and Labourers | - | - | 195 | 0.88 (0.77-1.02) | |
| Primary Production, Transportation, Manufacturing Managers | 10 | **1.11 (1.03-1.20)** | 635 | **1.09 (1.01-1.18)** | |
| Wood Working, Carpentry, and Processing, Sawmill | 15 | 0.95 (0.89-1.01) | 980 | 0.96 (0.90-1.02) | |
| Pulp and Paper Mill Machine Operators | - | - | 190 | 0.95 (0.83-1.10) | |
| **Metal Processing, Machinery and Construction** |  |  |  |  | |
| Metal and Mineral Processing | 20 | 0.96 (0.89-1.03) | 730 | 0.98 (0.91-1.05) | |
| Machinists and Tool Operators | 5 | 1.01 (0.92-1.11) | 400 | 1.00 (0.91-1.11) | |
| Machine Assemblers and Manufacturers | 5 | 1.05 (0.94-1.17) | 335 | 1.06 (0.95-1.18) | |
| Rubber and Plastic Products | - | - | 100 | 0.91 (0.75-1.11) | |
| Plumbers, Pipefitters, and Gas fitters | - | - | 340 | 0.97 (0.87-1.08) | |
| Painters | 10 | 0.95 (0.82-1.10) | 175 | 0.93 (0.81-1.08) | |
| Construction Managers and Supervisors | 15 | **1.07 (1.01-1.14)** | 1110 | **0.90 (0.84-0.97)** | |
| Construction Trades | 15 | **0.89 (0.83-0.96)** | 705 | 1.05 (0.99-1.11) | |
| **Transportation and Related** |  |  |  |  | |
| Transportation Equipment Operators | 20 | **0.91 (0.85-0.97)** | 905 | **0.90 (0.84-0.96)** | |
| Transportation Technicians and Maintenance Workers | 5 | 1.01 (0.89-1.13) | 280 | 1.00 (0.89-1.12) | |
| Motor Vehicle Repairers | 5 | **0.87 (0.80-0.95)** | 540 | **0.89 (0.82-0.97)** | |
| Vehicle Drivers | 20 | **0.92 (0.87-0.97)** | 1585 | **0.92 (0.88-0.97)** | |
| **Protective Services** |  |  |  |  | |
| Firefighters | - | - | 165 | **1.16 (1.00-1.35)** | |
| Armed Forces | 10 | 1.10 (0.95-1.26) | 190 | **1.14 (1.00-1.32)** | |
| Police Officers | 10 | **1.22 (1.09-1.36)** | 315 | **1.28 (1.14-1.42)** | |
| Other Protection Services | - | - | 565 | 0.97 (0.90-1.04) | |
| **Health and Personal Care** |  |  |  |  | |
| Dentists and Related | 5 | 1.08 (0.91-1.30) | 115 | 1.07 (0.90-1.28) | |
| General and Specialist Physicians | - | - | 305 | 0.91 (0.81-1.02) | |
| Registered Nurses, Supervisors and Aides | 5 | 0.98 (0.82-1.17) | 115 | 0.99 (0.83-1.18) | |
| Other Health Professionals and Related | 20 | 1.02 (0.94-1.11) | 575 | 1.02 (0.94-1.11) | |
| *^a^Hazard ratios (HR) adjusted for age, province, ethnicity, education, and marital status*  **Reference group: men employed in all other occupations except the occupation of interest *Case counts are rounded to base 5 using random rounding and counts <5 are not shown as per Statistics Canada reporting guidelines* | | | | | |

| **Supplementary Table 3. Hazard Ratios (HR) and Confidence Intervals (CI) for Prostate Cancer by Industry Group in the CanCHEC, stratified by age** | | | | |
| --- | --- | --- | --- | --- |
|  | **Age 25-49 years** | | **Age 50-74 years** | |
| **Industry Groups** | **Cases** | **HR^a^ (95% CI)** | **Cases** | **HR^a^ (95% CI)** |
| **Government and Administrative** |  |  |  |  |
| Federal Government Services | 35 | **1.11 (1.05-1.17)** | 1400 | **1.09 (1.03-1.15)** |
| Provincial and Territorial Government Services | 30 | 1.04 (0.97-1.10) | 990 | 1.01 (0.95-1.08) |
| Local and Other Government Services | 20 | 0.96 (0.91-1.02) | 1230 | 0.97 (0.92-1.02) |
| Business Services | 35 | 1.02 (0.98-1.06) | 2265 | 1.02 (0.97-1.06) |
| Finance Services | 20 | **1.13 (1.06-1.20)** | 1035 | 1.14 (1.07-1.21) |
| Educational Services | 25 | 1.02 (0.98-1.06) | 2620 | 0.96 (0.93-1.01) |
| **Natural Resources** |  |  |  |  |
| Agriculture Industries | 20 | **1.11 (1.06-1.16)** | 2525 | 1.04 (0.82-1.31) |
| Services Incidental to Agriculture | - | - | 70 | **1.11 (1.07-1.16)** |
| Forestry Services | - | - | 95 | 1.01 (0.83-1.23) |
| Logging Services | - | - | 310 | 0.97 (0.86-1.08) |
| Fishing and Trapping | 5 | 1.02 (0.90-1.15) | 245 | 1.03 (0.91-1.17) |
| Wood Products | 10 | 0.96 (0.87-1.05) | 455 | 0.98 (0.90-1.08) |
| Paper and Allied Products | 10 | 1.04 (0.96-1.13) | 570 | 1.06 (0.98-1.15) |
| Mining (Metal, Non-Metal, Coal Mines) | 10 | **0.88 (0.80-0.96)** | 455 | **0.88 (0.80-0.96)** |
| **Metal and Non-Metal Products** |  |  |  |  |
| Primary Metal Products | 10 | 0.98 (0.90-1.06) | 580 | 0.97 (0.90-1.06) |
| Fabricated Metal Products | 10 | 1.03 (0.95-1.11) | 615 | 1.04 (0.96-1.12) |
| Non-Metal Mineral Products | - | - | 275 | 1.05 (0.93-1.18) |
| Plastic Products | - | - | 200 | 1.15 (1.00-1.32) |
| Rubber Products | - | - | 75 | 0.89 (0.71-1.11) |
| Chemical Products | 5 | 0.98 (0.88-1.11) | 280 | 0.98 (0.87-1.10) |
| **Transportation and Trades** |  |  |  |  |
| Transportation Equipment and Repair | 25 | 0.98 (0.93-1.04) | 1105 | 0.99 (0.94-1.06) |
| Service Industries Incidental to Transportation | 5 | **0.86 (0.77-0.96)** | 320 | **0.86 (0.77-0.96)** |
| Other Transportation | 25 | 1.01 (0.97-1.06) | 2045 | 1.01 (0.96-1.05) |
| Automotive Vehicles, Parts and Accessories | 15 | 0.96 (0.90-1.02) | 1065 | 0.97 (0.91-1.03) |
| **Construction** |  |  |  |  |
| General Contracting and Development | 20 | 0.96 (0.91-1.03) | 980 | 0.98 (0.92-1.04) |
| Trade Contracting and Site Work | 40 | **0.93 (0.88-0.98)** | 1605 | **0.93 (0.89-0.98)** |
| Heavy Industrial Construction | 5 | 0.93 (0.85-1.02) | 415 | 0.92 (0.84-1.02) |
| Service Industries Incidental to Construction | 5 | 1.03 (0.93-1.15) | 335 | 1.03 (0.93-1.15) |
| **Technical and Informational Industries** |  |  |  |  |
| Telecommunications | 15 | **1.11 (1.04-1.18)** | 915 | **1.14 (1.06-1.21)** |
| Other Utility Industries (Electric, Gas, Water) | 15 | **1.10 (1.02-1.19)** | 680 | **1.10 (1.02-1.18)** |
| Printing, Publishing, and Allied Industries | 15 | 0.96 (0.87-1.05) | 415 | 0.96 (0.87-1.05) |
| **Health Service Industries** |  |  |  |  |
| Health and Social Services | 25 | 0.96 (0.91-1.01) | 1235 | 0.96 (0.90-1.01) |
| *^a^Hazard ratios (HR) adjusted for age, province, ethnicity, education, and marital status*  **Reference group: men employed in all other industries except the industry of interest *Case counts are rounded to base 5 using random rounding and counts <5 are not shown as per Statistics Canada reporting guidelines* | | | | |
